# Identification of a window of androgen sensitivity for somatic cell function in human fetal testis cultured ex vivo

**DOI:** 10.1186/s12916-022-02602-y

**Published:** 2022-10-20

**Authors:** Malene Lundgaard Riis, Gabriele Matilionyte, John E. Nielsen, Cecilie Melau, David Greenald, Kristine Juul Hare, Lea Langhoff Thuesen, Eva Dreisler, Kasper Aaboe, Pia Tutein Brenøe, Anna-Maria Andersson, Jakob Albrethsen, Hanne Frederiksen, Ewa Rajpert-De Meyts, Anders Juul, Rod T. Mitchell, Anne Jørgensen

**Affiliations:** 1 Department of Growth and Reproduction, Copenhagen University Hospital - Rigshospitalet, Blegdamsvej 9, 2100 Copenhagen, Denmark; 2 International Research and Research Training Centre in Endocrine Disruption of Male Reproduction and Child Health (EDMaRC), Blegdamsvej 9, Copenhagen, Denmark; 3 MRC Centre for Reproductive Health, The Queen’s Medical Research Institute, University of Edinburgh, 47 Little France Crescent, Edinburgh, EH16 4TJ UK; 4 Department of Obstetrics and Gynaecology, Copenhagen University Hospital - Hvidovre and Amager Hospital, Kettegård Alle 30, Hvidovre, Denmark; 5 Department of Gynaecology, Copenhagen University Hospital – Rigshospitalet, Blegdamsvej 9, DK-2100 Copenhagen, Denmark; 6 Department of Obstetrics and Gynaecology, Copenhagen University Hospital - Herlev and Gentofte Hospital, Borgmester Ib Juuls Vej 1, 2730 Herlev, Denmark; 7 Department of Clinical Medicine, University of Copenhagen, Copenhagen, Denmark

**Keywords:** Human fetal testis, Ex vivo culture, Reduced androgen exposure, Androgen sensitivity, Masculinization programming window

## Abstract

**Background:**

Reduced androgen action during early fetal development has been suggested as the origin of reproductive disorders comprised within the testicular dysgenesis syndrome (TDS). This hypothesis has been supported by studies in rats demonstrating that normal male development and adult reproductive function depend on sufficient androgen exposure during a sensitive fetal period, called the masculinization programming window (MPW). The main aim of this study was therefore to examine the effects of manipulating androgen production during different timepoints during early human fetal testis development to identify the existence and timing of a possible window of androgen sensitivity resembling the MPW in rats.

**Methods:**

The effects of experimentally reduced androgen exposure during different periods of human fetal testis development and function were examined using an established and validated human ex vivo tissue culture model. The androgen production was reduced by treatment with ketoconazole and validated by treatment with flutamide which blocks the androgen receptor. Testicular hormone production ex vivo was measured by liquid chromatography-tandem mass spectrometry or ELISA assays, and selected protein markers were assessed by immunohistochemistry.

**Results:**

Ketoconazole reduced androgen production in testes from gestational weeks (GW) 7–21, which were subsequently divided into four age groups: GW 7–10, 10–12, 12–16 and 16–21. Additionally, reduced secretion of testicular hormones INSL3, AMH and Inhibin B was observed, but only in the age groups GW 7–10 and 10–12, while a decrease in the total density of germ cells and OCT4^+^ gonocytes was found in the GW 7–10 age group. Flutamide treatment in specimens aged GW 7–12 did not alter androgen production, but the secretion of INSL3, AMH and Inhibin B was reduced, and a reduced number of pre-spermatogonia was observed.

**Conclusions:**

This study showed that reduced androgen action during early development affects the function and density of several cell types in the human fetal testis, with similar effects observed after ketoconazole and flutamide treatment. The effects were only observed within the GW 7–14 period—thereby indicating the presence of a window of androgen sensitivity in the human fetal testis.

**Supplementary Information:**

The online version contains supplementary material available at 10.1186/s12916-022-02602-y.

## Background

Testicular development of the initially bipotential gonad is directed by a signalling cascade that promotes the male pathway, while simultaneously antagonizing female signalling factors. Following the initial differentiation of the supporting cell lineage towards Sertoli cells, paracrine factors and steroid hormones secreted by the fetal Sertoli and Leydig cells contribute to the continued process of testicular development and masculinization of the fetus (reviewed in [1–3]). These processes have not yet been characterized in detail in human fetal development and although information from animal models provides essential insight there are important differences, particularly in relation to germ cell development and regulation of meiosis (reviewed in [2, 4]). Testosterone produced by the fetal Leydig cells plays a pivotal role as the main driver of fetal masculinization, although increasing evidence suggests that androgen precursors produced in the adrenal, liver and placenta via “backdoor” and 11-oxygenated steroidogenic pathways also contribute to the overall production of androgens in human fetuses as well as in the activation and masculinization of secondary sex characteristics [5–7].

The importance of sufficient androgen exposure during fetal development is evident from the clinical observations that male reproductive disorders included in the testicular dysgenesis syndrome (TDS) are most likely caused by subtle deficiencies in androgen production or action in the testis during fetal life [8, 9]. TDS is comprised of disorders that manifest either at birth (cryptorchidism, hypospadias) or in young adulthood (low sperm count, testicular germ cell cancer, primary hypogonadism) [8, 10, 11]. The fetal origin of cryptorchidism and hypospadias is intuitive, but the suggested fetal origin of adult-onset disorders, such as low sperm count, and hypogonadism was initially based on the finding of focal dysgenesis in the testes of the majority of adult men with TDS. The morphological alterations include abnormally shaped seminiferous tubules, Leydig cell nodules and Sertoli cell only (SCO) tubules, in which the Sertoli cells are occasionally visibly in the undifferentiated state [10–13], thereby suggesting abnormal early development of the testis. The resulting slightly impaired function of the somatic niche in the human fetal testis may disrupt the differentiation of germ cells and thus lead to the presence of arrested gonocytes which are the precursor cells of testicular germ cell cancer [8, 10]. While severe impairment of early testis development can result in overt dysgenesis and ensuing downstream effects as described in patients with differences of sex development [2, 13], the primary focus of the TDS hypothesis includes men in whom the dysgenetic changes are focal, often in an otherwise largely normal testis [13].

Normal masculinization of the fetus is dependent on testosterone production in the developing testes, and based on evidence from animal studies, this likely occurs during a specific sensitive period. The masculinization programming window (MPW) was discovered in rats and refers to a window of time during fetal life in which androgen action programs later development of male reproductive organs, including their adult size and function [14]. Reduced fetal testis testosterone levels during MPW induce focal dysgenesis in rats, followed by relatively normal testis differentiation, resembling TDS in humans [15]. Importantly, testicular dysgenesis in the rat model was only induced by androgen deficiency that occurs specifically within the MPW from embryonal day 15.5–18.5 [16]. Based on the results and extrapolations from animal models, it has been proposed that an equivalent human MPW exists most likely in the period between GW 8–14 [14, 17]. However, the existence of a human MPW and its timing has yet to be identified. Therefore, this study aimed to examine whether an androgen-sensitive window can be identified during early human fetal testis development. Specifically, the study focused on the effects of androgen deficiency induced by treatment with ketoconazole or flutamide on somatic cell function and on germ cell density and maturation during the presumed critical window. Since it is not possible to conduct such a study in humans in vivo, an established and extensively validated ex vivo culture model of human fetal testis was used in this study [12, 18, 19].

## Methods

### Collection of human fetal gonads and ethical approval in Denmark

Human fetal testes were isolated from material available following elective termination of pregnancy during the 1^st^ trimester at the Department of Gynaecology at Copenhagen University Hospital (Rigshospitalet), Hvidovre Hospital and Herlev Hospital, Denmark. The regional ethics committee approved this study (permit number H-1-2012-007, including amendments 48801, 50662, 55184, 64377 and 68831), and women gave their informed written and oral consent. None of the terminations was due to pathology of pregnancy or fetal abnormality. The embryos/fetuses included in this study were between 7 and 12 GW, with fetal age determined by scanning crown-rump length and by evaluation of foot length [20]. The fetal tissues were dissected in ice-cold PBS and the isolated fetal gonads were immediately set up in ex vivo cultures. In total, 40 specimens of 1^st^-trimester testis tissue were used for the current study.

### Collection of human fetal gonads and ethical approval in the UK

Human fetal testes were isolated from material available following elective termination of pregnancy during the 2^nd^ trimester (13–21 GW). Women gave written informed consent in accordance with national guidelines, and ethical approval was obtained from the Lothian Research Ethics Committee (LREC08/S1101/1). No terminations were related to fetal abnormalities. In addition, fetal tissue was provided by the Human Developmental Biology Resource (www.hdbr.org). After dissection, the fetal testis was placed immediately into ice-cold media containing Liebowitz L-15 with glutamine, 10% fetal bovine serum, 1% penicillin/streptomycin and 1% non-essential amino acids (all Sigma, Poole, UK) before ex vivo culture. Testis tissues from 11 2^nd^-trimester fetuses were used for ex vivo culture experiments.

### Ex vivo gonad tissue culture and treatments

Human fetal testes were cultured ex vivo in hanging drops as described previously [12, 18, 19], with a few modifications. For the GW 7–12 samples collected in Copenhagen, the aborted tissue was transported to the laboratory where it was dissected and thereafter immediately set up in culture (with culture media containing FBS). For the GW 13–21 samples collected in Edinburgh, the aborted tissue was dissected and transported (in L-15 media) to the laboratory typically within a few hours after collection, before it was set up in culture. Prior to culture set-up, all gonads were divided into approximately 1-mm^3^ fragments with at least one piece from each embryo/fetus used as vehicle control. Each tissue piece was cultured in a 40-µl medium for 14 days. The medium composition was MEMα medium supplemented with 1× MEM non-essential amino acids, 2 mM sodium pyruvate, 2 mM l-glutamine, 1 × insulin, transferrin and selenium (ITS) supplement, 1 × penicillin/streptomycin and 10% fetal bovine serum (FBS). All cell media and supplements were from Gibco (Nærum, Denmark), except ITS (Sigma-Aldrich, Brøndby, Denmark). Fragments of gonads were cultured at 37°C under 5% CO_2_ with complete medium change every 48 h. Culture media were collected every 48 h and pooled throughout the 14-day culture period for each tissue fragment and were subsequently used for measurement of hormone production. To manipulate androgen exposure, fetal testis tissue was cultured in a medium containing either 10^−6^ M ketoconazole (Sigma-Aldrich, UC280) to reduce androgen production, 1 IU/ml hCG (Prospec, hor-250-a) to stimulate steroidogenesis or 10^−6^ M flutamide (Sigma-Aldrich, F9397) to block the androgen receptor with respecting treatments administered a total of 6 times during the 14 days of the culture period. Human fetal testis samples from GW 7–21 treated with ketoconazole and hCG were subsequently divided into four groups according to developmental age (in gestational week + days): GW 7–10 (GW 7+2 days–9+6 days), 10–12 (GW 10+0 days–11+6 days), 12–16 (GW 12+0 days–15+6 days) and 16–21 (GW 16+0 days–21+0 days). Additionally, samples from GW 7–12 (GW 7+4 days–11+5 days) were treated with flutamide and ketoconazole in a subsequent series of experiments. Ketoconazole and flutamide were dissolved in dimethyl sulfoxide vehicle (DMSO, 0.1%) (Sigma-Aldrich) and hCG was diluted in PBS with 0.1% BSA. At the end of the ex vivo culture period, the testis tissue fragments were formalin fixed.

### Immunohistochemistry

The testicular samples were dehydrated, paraffin-embedded and sectioned (4 µm) using standard procedures. Serial sections of each sample were used for immunohistochemistry (IHC) as previously described for formalin-fixed samples [21], except that tissue sections were subjected to heat-induced antigen retrieval buffer in a microwave. Endogenous peroxidase activity was blocked with 1% (v/v) H_2_O_2_ in MeOH for 30 min. Sections were then incubated with either (A) 0.5% milk powder diluted in Tris-buffered saline (TBS) or (B) 5% BSA (w/v) in ImmPRESS horse serum (20% v/v) (Vector Laboratories, Burlingame, CA) for 30 min to minimize cross-reactivity. Primary antibodies were incubated overnight at 4°C followed by 1 h at room temperature. Sections were incubated for 30 min with the appropriate ImmPRESS HPR (peroxidase, Vector Laboratories, Burlingame, CA) secondary antibody diluted in normal serum. Primary antibodies, dilutions and retrieval buffers are listed in Table 1. Visualization was performed using ImmPACT AEC peroxidase substrate (Vector Laboratories, Burlingame, CA). Sections were washed in Tris-buffered saline between each step in this protocol. Negative controls were included and processed with the primary antibody replaced by the dilution buffer alone, none of which showed staining. Sections were counterstained with Mayer’s haematoxylin before mounting with Aquatex (Merck, Damstadt, Germany). Sections were initially evaluated on a Nikon Microphot-FXA microscope and then by scanning slides on a NanoZoomer 2.0 HT (Hamamatsu Photonics, Herrsching am Ammersee, Germany) followed by analysis using the NDPview software, version 1.2.36 (Hamamatsu Photonics, Herrsching am Ammersee, Germany).

**Table 1:** Antibodies, dilutions and retrieval buffers used

**Antibody**	**Dilution**	**Retrieval buffer**	**Species**	**Supplier**	**Number**
AR	1:75	TEG	Mouse	Santa Cruz	Sc-7305
BrdU	1:100	CIT	Mouse	Dako	M0744
CYP11A1	1:500	CIT	Rabbit	Sigma	HPA016436
cPARP	1:500	CIT	Rabbit	Cell Signaling	5625
MAGE-A4	1:250	TEG	Mouse	Gift from Prof. Spagnoli	NA
OCT4	1:250	TEG	Mouse	Santa Cruz	Sc-5279
SOX9	1:800	CIT	Rabbit	Millipore	AB5535

Antigen retrieval was conducted by microwaving the sections in the indicated retrieval buffer for 1 min at 750W and 15 min at 350W. TEG buffer: 10 mM Tris, 0.5 mM EGTA, pH 9.0; citrate (CIT) buffer: 10 mM, pH 6.0. *NA* not available

### BrdU incorporation

Before the end of the ex vivo culture period, tissue fragments were cultured with BrdU labelling agent (Life Technologies, Nærum, Denmark) diluted 1:10 in media for 6 h to allow for the detection of proliferating cells in the tissue. After 6 h, tissue fragments were formalin fixated and paraffin-embedded as described above. Proliferating cells were visualized by immunohistochemical analysis using a BrdU antibody (Table 1) as described in the immunohistochemistry section.

### Quantification of stained cells

To evaluate the IHC staining, the stained cells were quantified per area of tissue using one entire tissue section per sample, thereby determining the density of the specific cell type. The area was calculated using the NDPview software (Hamamatsu Photonics, Herrsching am Ammersee, Germany). Gonocytes were identified based on OCT4 staining and pre-spermatogonia by MAGE-A4 staining. Tissue samples from at least 5 embryos/fetuses were included in quantifications of stained cells.

### Steroid hormone measurements by liquid chromatography-tandem mass spectrometry (LC-MS/MS)

Steroid hormone levels in culture media following ex vivo culture were measured as described previously [12, 19]. Steroid hormone levels (nM concentrations) were measured using a sensitive isotope-dilution TurboFlow-LC-MS/MS method [22]. This clinically validated analysis package includes measurement of the androgens: testosterone, androstenedione and dehydroepiandrosterone sulphate (DHEAS); glucocorticoids: cortisone and cortisol; and the steroidogenic intermediates: 11-deoxycortisol, 17-hydroxyprogesterone (17-OHP), progesterone and corticosterone. All measured steroids, except estrone sulphate, are reported in this study. The method was modified for measurement in culture media. In brief, samples were analysed in four batches between 2018 and 2021. For all batches, two blanks (water), two un-spiked media controls, two spiked media controls with a mixture of native steroid standards in low concentrations and two spiked media controls with the native steroid standards in high concentrations were used as method controls, while standards prepared in media were used for calibration curves. All collected media samples were diluted four times in culture media prior to analysis. A few samples were re-analysed for testosterone after additional sample dilution. For all analytical batches included in this study, the relative standard deviation (RSD) was <14% for all analytes in low spike levels except for 17-hydroxyprogesterone (<20%) and progesterone (<17%), whereas RSD was <6.5% for all analytes in the controls spiked in high level.

### INSL3 measurements by liquid chromatography-tandem mass spectrometry (LC-MS/MS)

INSL3 concentration in culture media from ex vivo culture was determined by LC-MS/MS as previously described [23]. The limits of detection and quantification were 0.03 and 0.15 µg/l, respectively, and the intra-assay variation was <10%. INSL3 calibrants were created as previously described, except that the INSL3 stocks were prepared in culture media.

### AMH measurements by ELISA

AMH concentration in culture media from ex vivo culture was measured by ELISA using the Beckman Coulter enzyme immunometric assay as previously described [12]. In brief, the collected media samples were diluted 1:10 in culture media prior to analysis, with additional sample dilution (1:25 and 1:50) necessary for 19% and 5% of the samples, respectively. The detection limit of the AMH assay was 0.14 pmol/l and the intra-assay variation was <9% in the total measurement range.

### Inhibin B measurements by ELISA

Inhibin B concentration in culture media from ex vivo culture was measured by ELISA using the Beckman Coulter Inhibin B Generation II enzyme immunometric assay Kit as previously described [12]. In brief, the collected media samples were diluted 1:50 in culture media prior to analysis, with additional sample dilution (1:75 and 1:100) necessary for 2% and 1% of the samples, respectively. The detection limit of the Inhibin B assay was 3 pg/ml and the inter-assay variation was <10% in the total measurement range.

### Statistical analysis

Statistical analysis was performed using GraphPad Prism Software. Data are presented as mean ± SEM. Student’s paired (two-tailed) *t*-test was used. Asterisks indicate statistical significance with *p<0.05, **p<0.01, ***p<0.001 and ****p<0.0001. The number of replicates is specified for each experiment/analysis in the figure legends.

## Results

### Manipulation of steroidogenesis in ex vivo culture of human fetal testis

Previous studies have demonstrated that steroidogenic activity of human fetal testis tissue was maintained and can be manipulated in our established ex vivo culture model [12, 18, 19]. Thus, it was initially established that the androgen production could be inhibited and induced following 2-week treatment with ketoconazole (10^−6^ M) and hCG (1 IU/ml), respectively, in human fetal testis tissue from 1^st^ and 2^nd^ trimesters cultured ex vivo. As expected, treatment with ketoconazole resulted in reduced androgen production, while hCG treatment increased the secretion of all measured steroid hormone metabolites compared with vehicle control treated samples (data not shown).

**Fig. 1:**
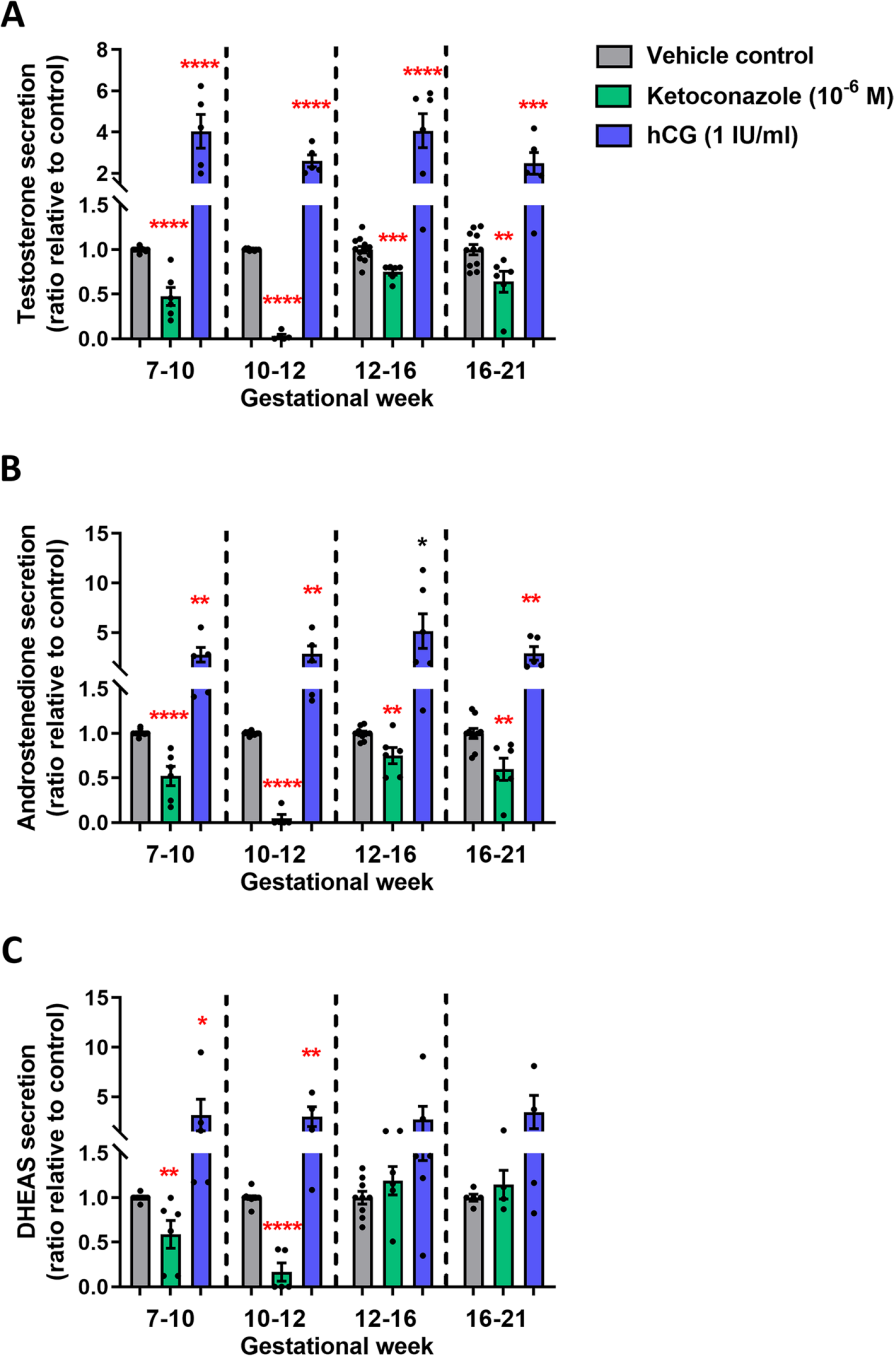
Manipulation of androgen production in ex vivo culture of human fetal testes from gestational weeks 7–21. Quantification of **A** testosterone, **B** androstenedione and **C** DHEAS produced in the fetal testis tissue ex vivo cultures and secreted to the media droplets following treatment with ketoconazole (10^−6^ M) and hCG (1 IU/ml) for 14 days. Media were collected every 48 h throughout the 14-day culture period and were pooled for each individual tissue piece. Androgens were measured by LC-MS/MS and are shown as ratios compared to the mean of the corresponding vehicle controls (from the same fetus). Samples are divided into four groups according to developmental timepoints corresponding to gestational weeks (GW) 7–10, 10–12, 12–16 and 16–21. Values represent mean ± SEM, with *N*=5–6 for each age group and treatment. Significant difference compared to vehicle control, ****p<0.0001, ***p<0.001, **p<0.01 and *p<0.05

**Fig. 2:**
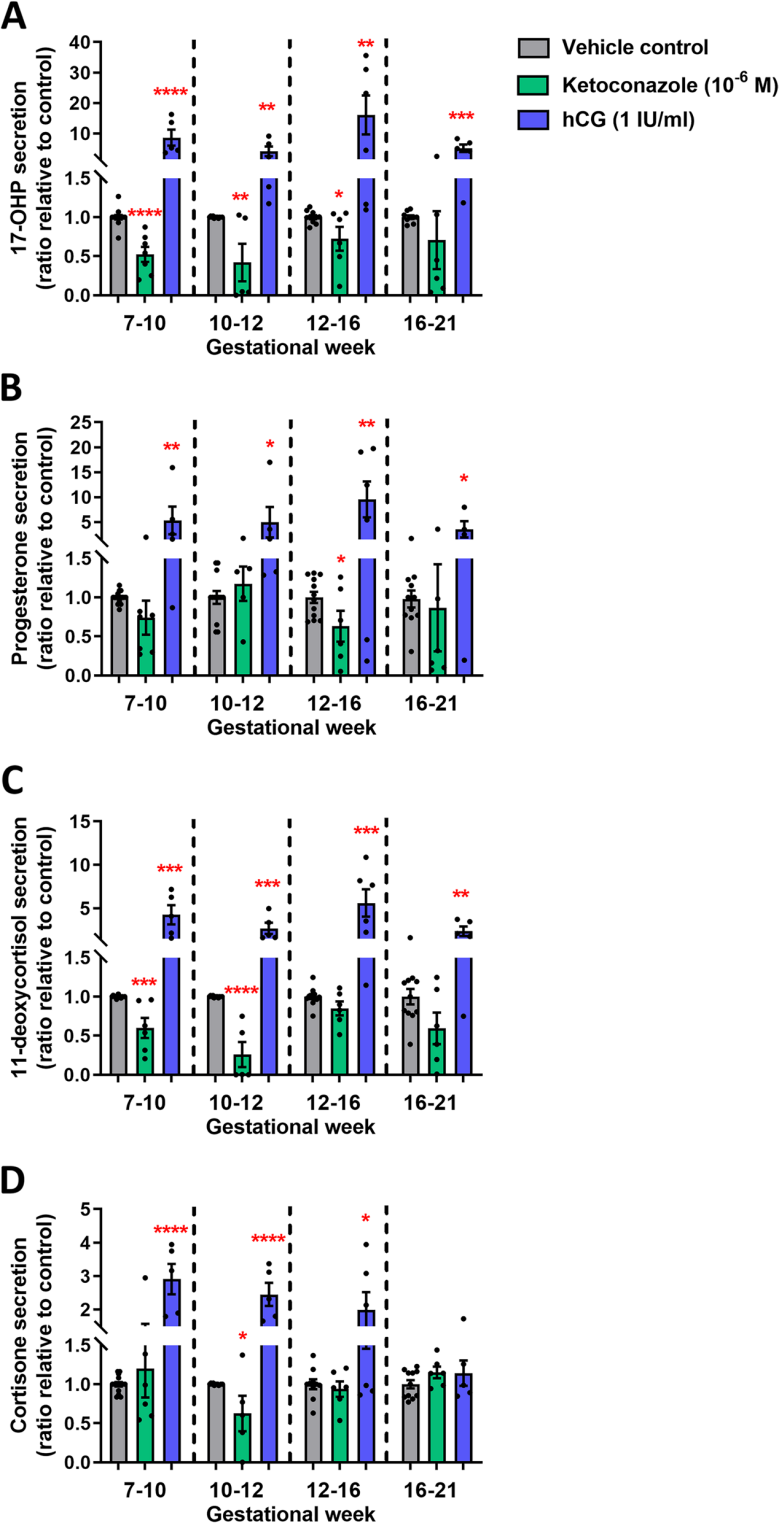
Manipulation of steroidogenesis in ex vivo culture of human fetal testes from gestational weeks 7–21. Quantification of **A** 17-OHP, **B** progesterone, **C** 11-deoxycortisol and **D** cortisone produced in the fetal testis tissue ex vivo cultures and secreted to the media droplets following treatment with ketoconazole (10^−6^ M) and hCG (1 IU/ml) for 14 days. Media were collected every 48 h throughout the 14-day culture period and were pooled for each individual tissue piece. Steroid metabolites were measured by LC-MS/MS and are shown as ratios compared to the mean of the corresponding vehicle controls (from the same fetus). Samples are divided into four groups according to developmental timepoints corresponding to gestational weeks (GW) 7–10, 10–12, 12–16 and 16–21. Values represent mean ± SEM, with *N*=5–6 for each age group and treatment. Significant difference compared to vehicle control, ****p<0.0001, ***p<0.001, **p<0.01 and *p<0.05

The ketoconazole-mediated inhibition of androgen production was significant in all of the four developmental age groups (Fig. 1A–C), except DHEAS in GW 12–16 and 16–21. Importantly, the production of testosterone was reduced in all age groups after ketoconazole treatment (GW 7–10: 72%, *p*<0.0001, GW 10–12: 188%, *p*<0.0001, GW 12–16: 29%, *p*<0.01 and GW 16–21: 44%, *p*<0.001) (Fig. 1A). A similar pattern was seen for the reduction in androstenedione production following ketoconazole treatment (Fig. 1B), while DHEAS levels were only reduced in the GW 7–10 and GW 10–12 groups (Fig. 1C). Treatment with hCG (1 IU/ml) significantly stimulated androgen production (testosterone, androstenedione and DHEAS) in all age groups (Fig. 1A–C), except DHEAS which was not stimulated in the GW 12–16 and GW 16–21 groups (Fig. 1C). The levels of glucocorticoid and mineralocorticoid metabolites overall increased following hCG treatment in all four age groups (Fig. 2), while ketoconazole treatment did not systematically reduce the level of glucocorticoid and mineralocorticoid metabolites, although the level of 17-OHP (GW 7–10, GW 10–12 and GW 12–16) and 11-deoxycortisol (GW 7–10 and GW 10–12) was reduced (Fig. 2). Thus, androgen production could be manipulated in both directions in the ex vivo cultured human fetal testis tissue following treatment with ketoconazole and hCG.

### Effects of altered androgen production on Leydig cells

**Fig. 3:**
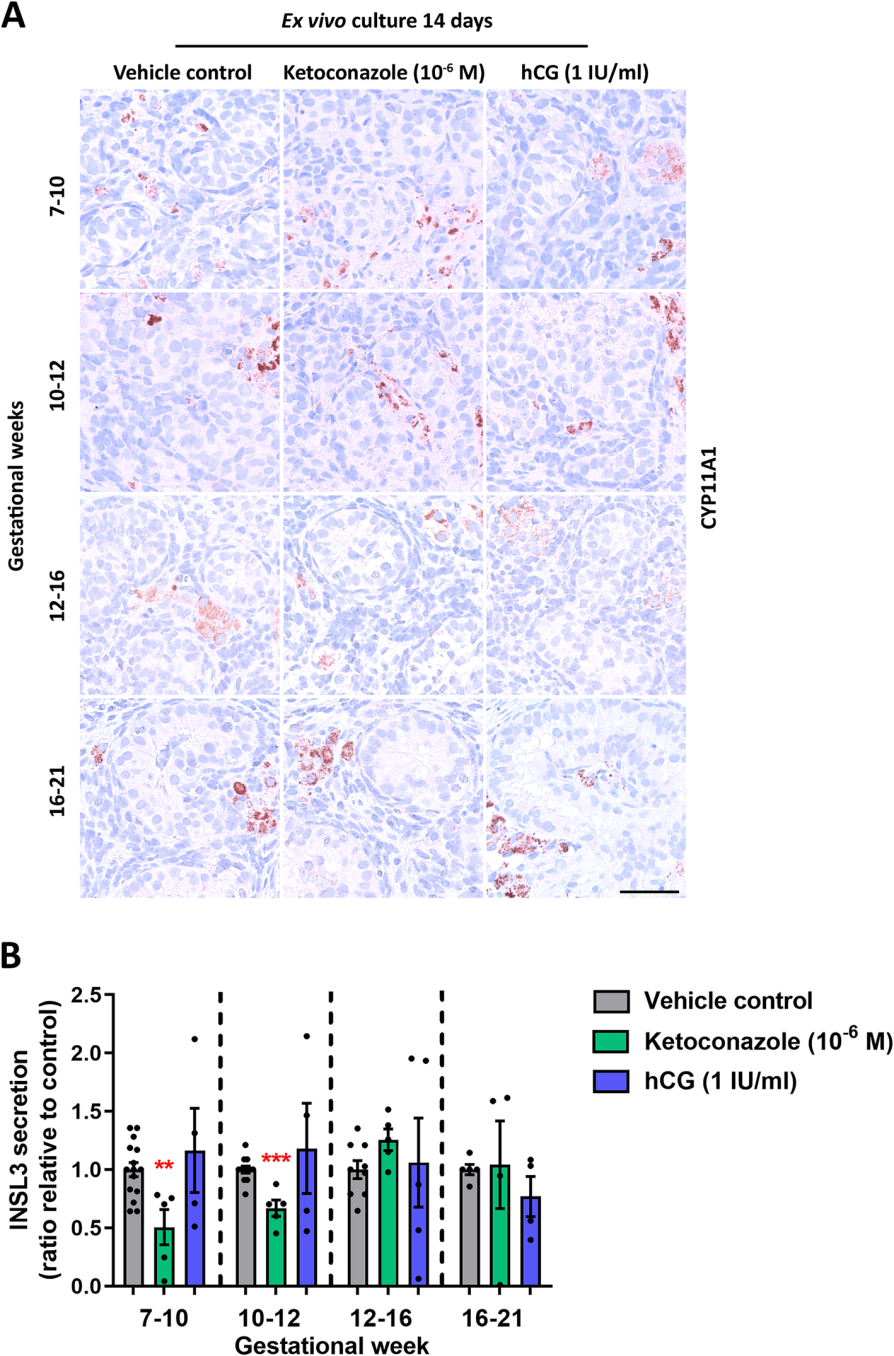
Effects of manipulating androgen production in ex vivo cultured human fetal testis tissue on CYP11A1 expression and INSL3 production. **A** Expression pattern of the Leydig cell marker CYP11A1 in fetal testis samples treated with ketoconazole (10^−6^ M) and hCG (1 IU/ml) for 2 weeks in ex vivo culture. Images representative for the expression in each age group and treatment. Counterstaining with Mayer haematoxylin; the scale bar corresponds to 50 µm. **B** Quantification of INSL3 secretion by the ex vivo cultured fetal testis tissue following treatment with ketoconazole (10^−6^ M) and hCG (1 IU/ml) for 14 days. Media were collected every 48 h throughout the 14-day culture period and were pooled for each individual tissue piece. INSL3 was measured by LC-MS/MS and is shown as ratios compared to the mean of the corresponding vehicle controls (from the same fetus). Samples are divided into four groups according to developmental timepoints corresponding to gestational weeks (GW) 7–10, 10–12, 12–16 and 16–21. Values represent mean ± SEM, with *N*=5–6 for each age group and treatment. Significant difference compared to vehicle control, ***p<0.001 and ** *p*<0.01

Subsequently, additional effects on fetal Leydig cells were examined following treatment of the ex vivo cultured fetal testis tissue with ketoconazole (10^−6^ M) and hCG (1 IU/ml). The expression pattern of CYP11A1 and CYP17A1 showed no apparent difference following treatment with either ketoconazole or hCG in any of the age groups (Fig. 3A and data not shown). Also, treatment with ketoconazole and hCG did not overall result in changes in testicular morphology or seminiferous cord structure (Fig. 3A) and no apparent changes in the level of apoptotic (cPARP^+^) or proliferating cells (BrdU^+^) were observed following treatment with ketoconazole or hCG (Additional file 1: Fig. S1 and data not shown). The androgen receptor (AR) was expressed in Leydig cells and peritubular myoid (PTM) cells during the investigated developmental period, while no or very few Sertoli cells were AR^+^. The expression pattern of AR was not altered following treatment with ketoconazole or hCG (data not shown). In contrast, the secretion of INSL3 by the Leydig cells was significantly reduced following treatment with ketoconazole, although only in the age groups GW 7–10 (65%, *p*<0.01) and GW 10–12 (40%, *p*<0.001), while no effect on INSL3 production was seen in the GW 12–16 and GW 16–21 groups (Fig. 3B). No changes in INSL3 secretion were found after hCG treatment in any of the four age groups (Fig. 3B).

### Effects of altered androgen production on Sertoli cell function

**Fig. 4:**
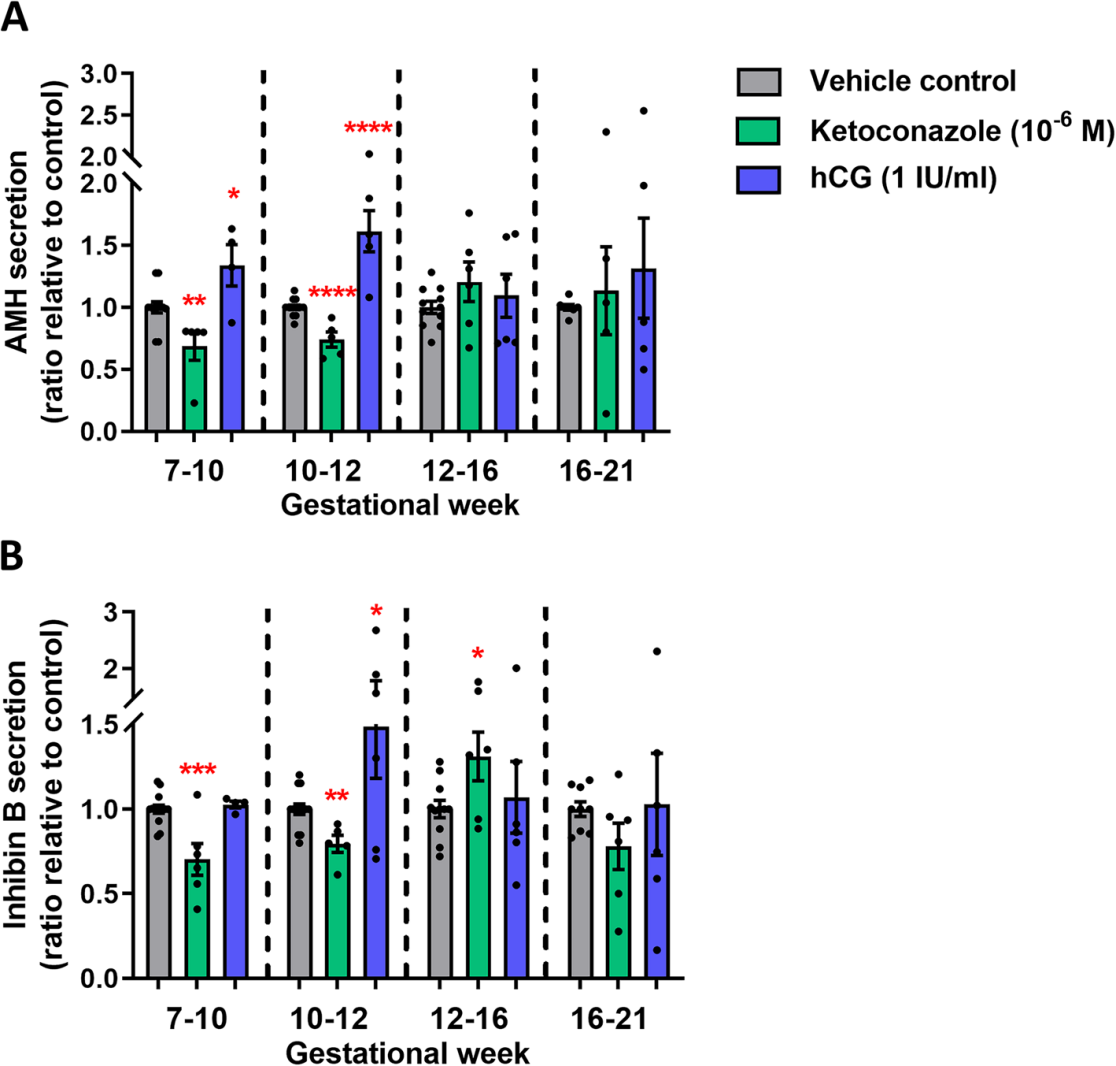
Effects of manipulating androgen production in ex vivo cultured human fetal testis tissue on Sertoli cell function. Quantification of **A** AMH and **B** Inhibin B secretion from the Sertoli cells by the ex vivo cultured fetal testis tissue following treatment with ketoconazole (10^−6^ M) and hCG (1 IU/ml) for 14 days. Media were collected every 48 h throughout the 14-day culture period and were pooled for each individual tissue piece. AMH and Inhibin B were measured by ELISA and are shown as ratios compared to the mean of the corresponding vehicle controls (from the same fetus). Samples are divided into four groups according to developmental timepoints corresponding to gestational weeks (GW) 7–10, 10–12, 12–16 and 16–21. Values represent mean ± SEM, with *N*=5–6 for each age group and treatment. Significant difference compared to vehicle control, ****p<0.0001, ***p<0.001, **p<0.01 and *p<0.05

Next, effects on Sertoli cells were examined following ketoconazole-mediated inhibition of androgen production in the ex vivo cultured human fetal testes. The expression pattern of the Sertoli cell marker SOX9 showed no overall difference following treatment in any of the age groups with either ketoconazole or hCG, except possibly a slightly reduced expression of SOX9 after ketoconazole treatment in the GW 7–10 and GW 10–12 groups (Additional file 2: Fig. S2). Sertoli cell function was assessed by secretion of AMH and Inhibin B to the culture media. AMH production was reduced following ketoconazole treatment in the age groups GW 7–10 (37%, *p*<0.01) and GW 10–12 group (30%, *p*<0.0001), while hCG treatment increased AMH secretion in these groups, GW 7–10 (29%, *p*<0.05) and GW 10–12 group (46%, *p*<0.0001) (Fig. 4A). No effects of ketoconazole or hCG treatment on AMH secretion were found in the GW 12–16 and GW 16–21 groups (Fig. 4A). Similar effects were found for Inhibin B secretion, including reduced levels after ketoconazole treatment in the age groups GW 7–10 (35%, *p*<0.001) and GW 10–12 group (23%, *p*<0.01), and increased Inhibin B level in the GW 10–12 group (40%, *p*<0.05) after hCG treatment (Fig. 4B). No effects of hCG treatment on Inhibin B secretion were observed in the GW 7–10, GW 12–16 and GW 16–21 groups, nor after ketoconazole treatment in the GW 16–21 group. However, an unexpected increase in Inhibin B secretion was found in the GW 12–16 group after ketoconazole treatment (26%, *p*<0.05) (Fig. 4B).

### Effects of altered androgen production on germ cell numbers

**Fig. 5:**
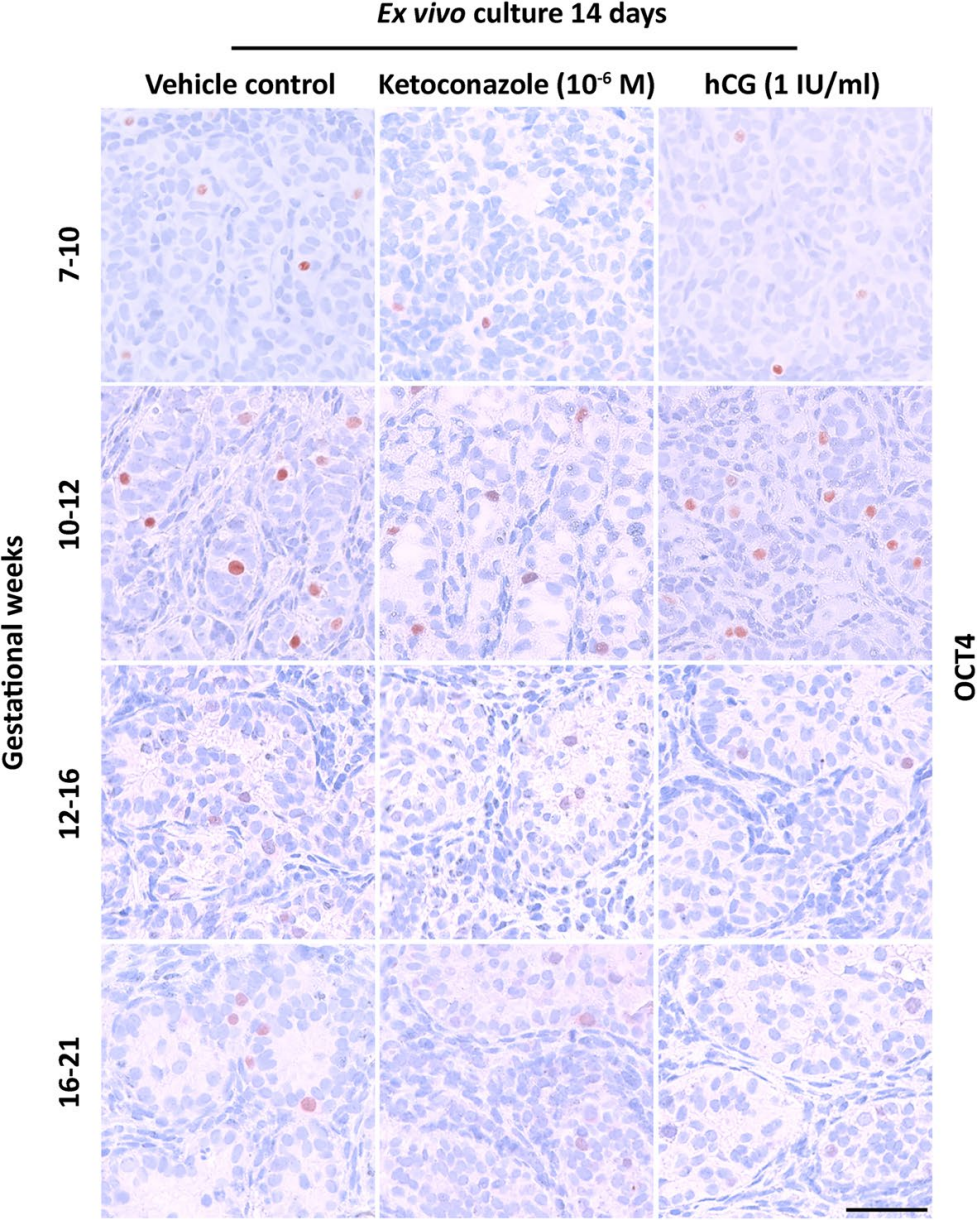
Effects of manipulating androgen production in ex vivo cultured human fetal testis tissue on the expression of the germ cell marker OCT4. Expression of the germ cell markers OCT4 in fetal testis samples treated with ketoconazole (10^−6^ M) and hCG (1 IU/ml) for 2 weeks in ex vivo culture. Images representative for the expression in each age group and treatment. Counterstaining with Mayer haematoxylin; the scale bar corresponds to 50 µm

**Fig. 6:**
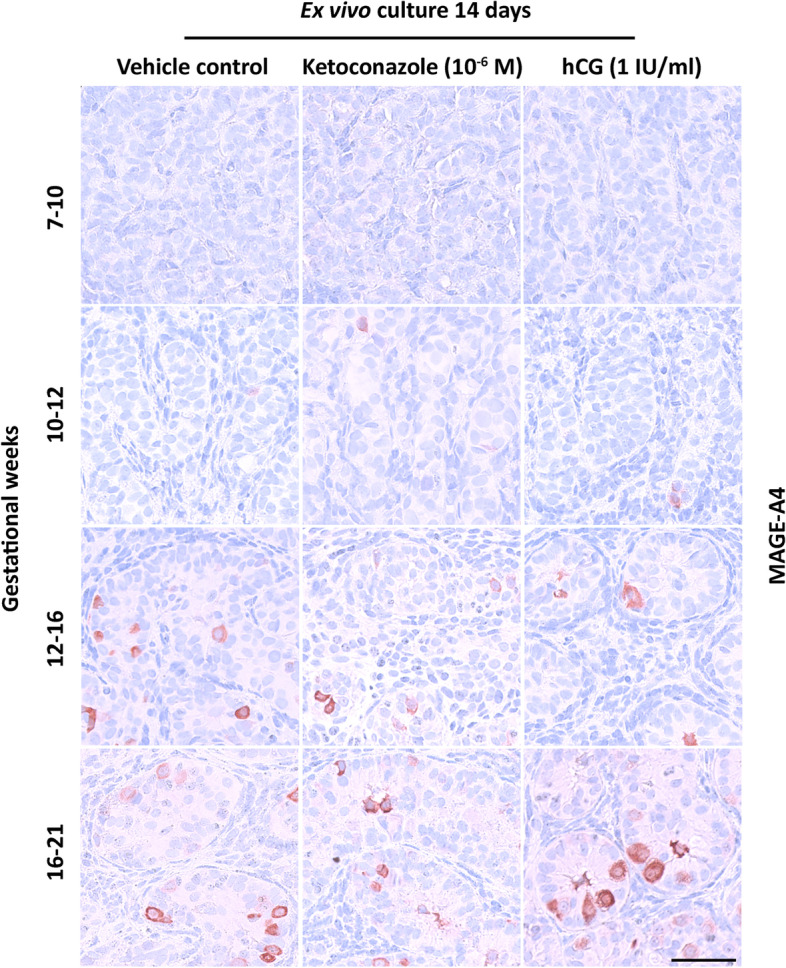
Effects of manipulating androgen production in ex vivo cultured human fetal testis tissue on the expression of germ cell marker MAGE-A4. Expression of the germ cell marker MAGE-A4 in fetal testis samples treated with ketoconazole (10^−6^ M) and hCG (1 IU/ml) for 2 weeks in ex vivo culture. Images representative for the expression in each age group and treatment. Counterstaining with Mayer haematoxylin; the scale bar corresponds to 50 µm

**Fig. 7:**
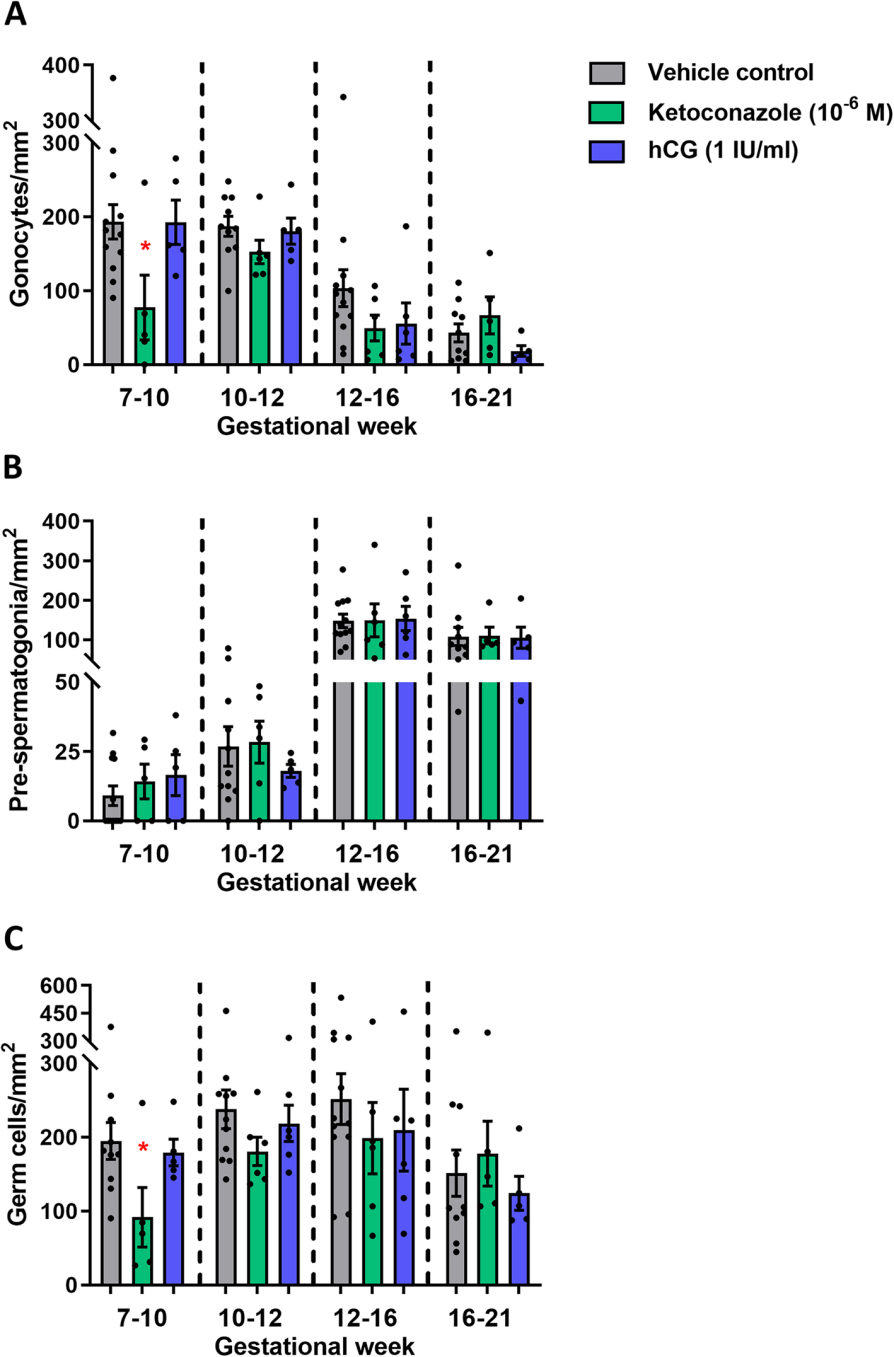
Effects of manipulating androgen production in ex vivo cultured human fetal testis tissue on germ cell density. Quantification of **A** gonocytes, **B** pre-spermatogonia and **C** total germ cell number per mm^2^ (density) in the ex vivo cultured fetal testis tissue following treatment with ketoconazole (10^−6^ M) and hCG (1 IU/ml) for 14 days. Gonocytes were determined as the number of OCT4^+^ cells per mm^2^, pre-spermatogonia as the number of MAGE-A4^+^ cells per mm^2^ and total germ cell number as the combined number of OCT4^+^ and MAGE-A4^+^ cells per mm^2^. Samples are divided into four groups according to developmental timepoints corresponding to gestational weeks (GW) 7–10, 10–12, 12–16 and 16–21. Values represent mean ± SEM, with *N*=5–6 for each age group and treatment. Significant difference compared to vehicle control, *p<0.05

Effects on germ cells were examined by immunohistochemical staining of tissue from human fetal testis cultures after manipulation of androgen production. OCT4 was used as a marker for fetal gonocytes and MAGE-A4 as a marker for pre-spermatogonia. Since differentiation from gonocyte to pre-spermatogonia occurs in an asynchronous manner during the examined developmental period both markers were used. As expected, an overall higher number of OCT4^+^ gonocytes was seen in the GW 7–10 and 10–12 groups (Fig. 5), while MAGE-A4^+^ pre-spermatogonia were more frequently observed in GW 12–16 and 16–21 groups (Fig. 6). The number of gonocytes (OCT4^+^), pre-spermatogonia (MAGE-A4^+^) and total germ cells per area was quantified to estimate cell density. No differences were found after ketoconazole treatment, except in the GW 7–10 group. Here, ketoconazole treatment resulted in a reduced density of OCT4^+^ cells/mm^2^ (86%, *p*<0.05) (Fig. 7A) and a reduced density of total germ cells/mm^2^ (72%, *p*<0.05) (Fig. 7C). There was no difference in the density of pre-spermatogonia (MAGE-A4^+^) after ketoconazole treatment in any of the age groups (Fig. 7B) and no differences in any of the germ cell populations were found after hCG treatment in the four age groups (Fig. 7A–C).

### Effects of reduced androgen action via blocking of the androgen receptor

**Fig. 8:**
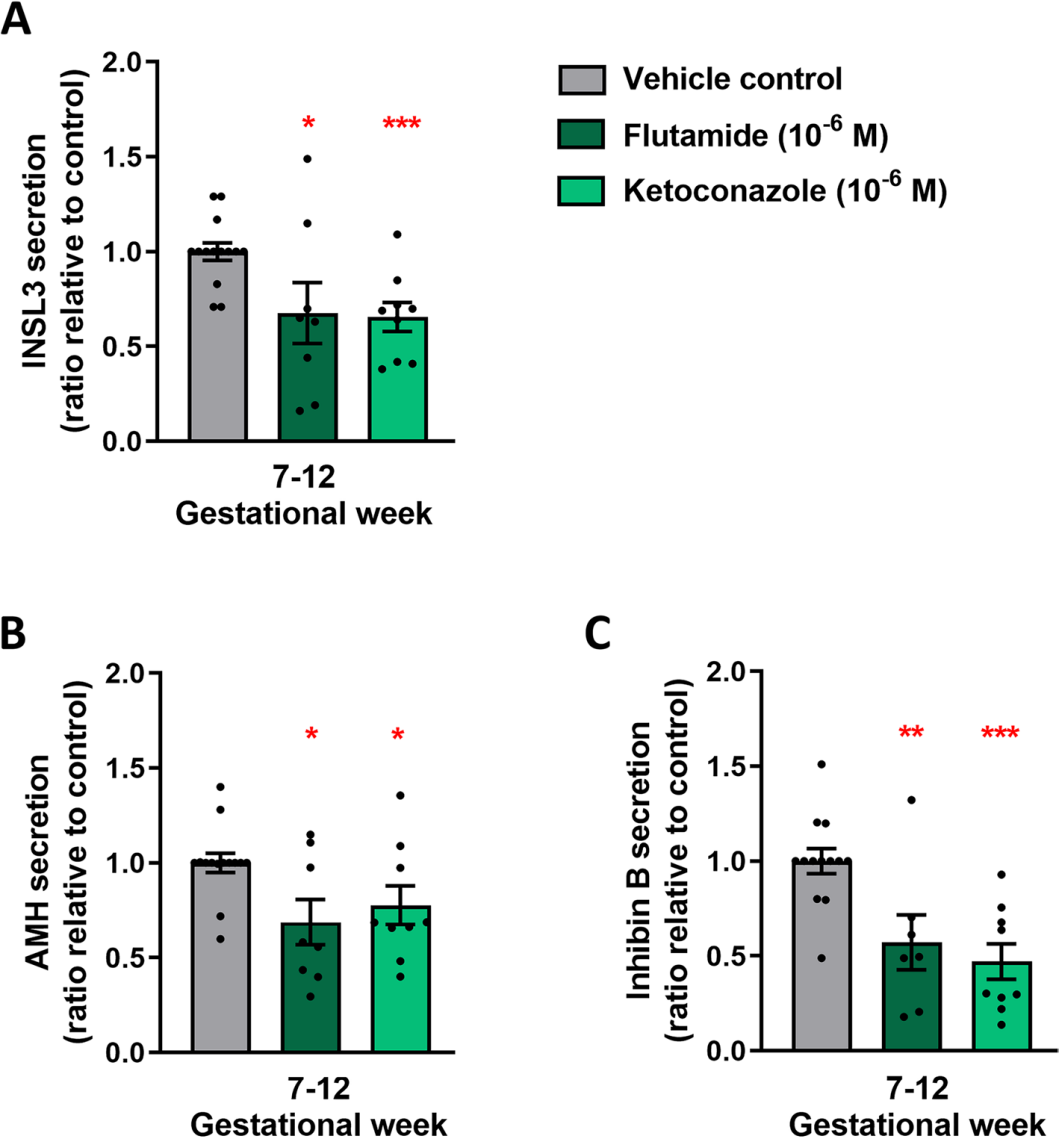
Effects of reduced androgen exposure via blocking of the androgen receptor in ex vivo culture of human fetal testes. Quantification of **A** INSL3, **B** AMH and **C** Inhibin B secretion by ex vivo cultured fetal testis tissue from GW 7–12 following treatment with flutamide (10^−6^ M) and ketoconazole (10^−6^ M) for 14 days. Media were collected every 48 h throughout the 14-day culture period and were pooled for each individual tissue piece. INSL3 was measured by LC-MS/MS, while AMH and Inhibin B were measured by ELISA. Results are shown as the ratio compared to the mean of the corresponding vehicle controls (from the same fetus). Values represent mean ± SEM, with *N*=13–14 (vehicle control), *N*=7–8 (flutamide) and *N*=9 (ketoconazole). Significant difference compared to vehicle control, ***p<0.001, **p<0.01 and *p<0.05

**Fig. 9:**
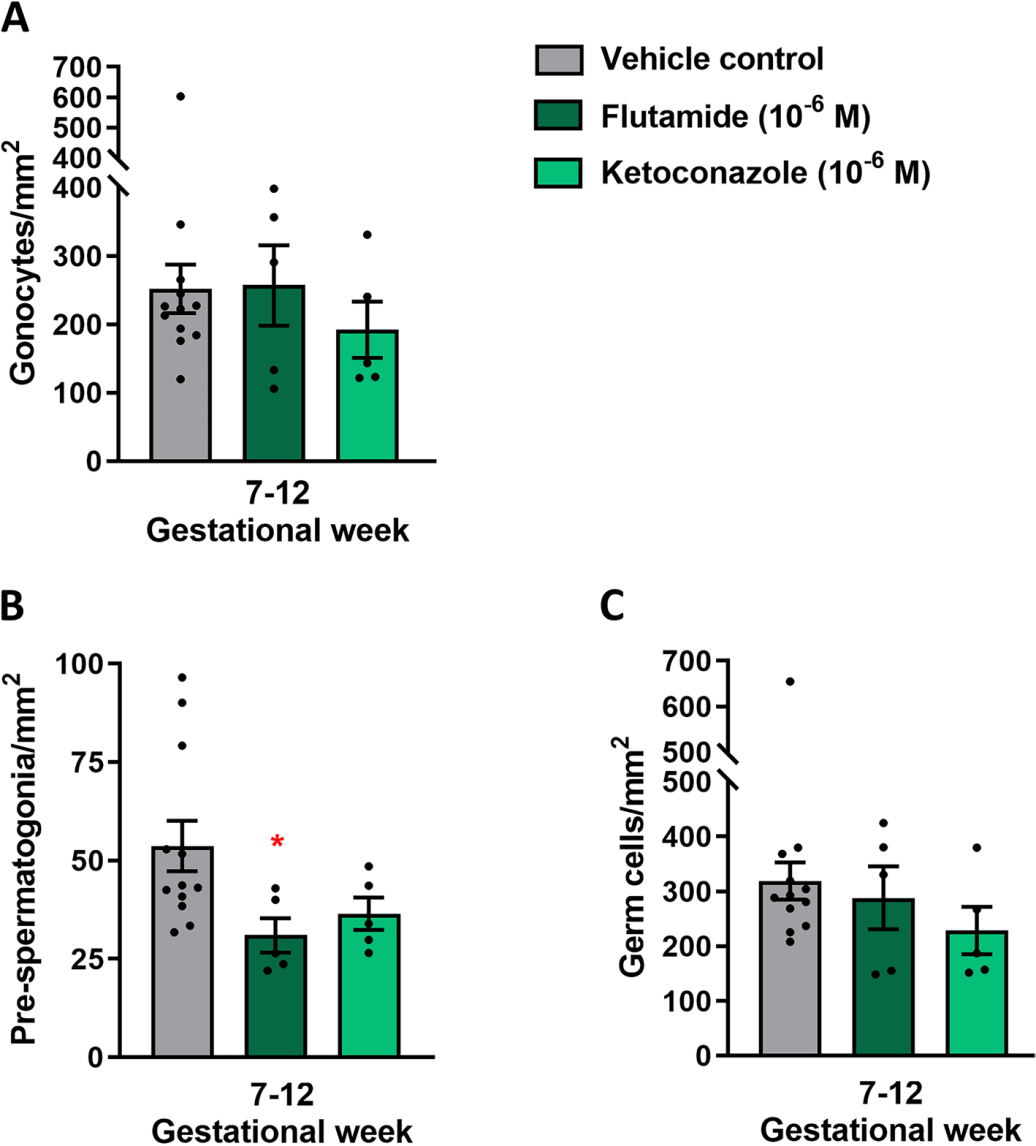
Effects of reduced androgen exposure via blocking of the androgen receptor in ex vivo culture of human fetal testes on germ cell density. Quantification of **A** gonocytes, **B** pre-spermatogonia and **C** total germ cell number per mm^2^ in the ex vivo cultured fetal testis tissue from GW 7–12 following treatment with flutamide (10^−6^ M) for 14 days. Gonocytes were determined as the number of OCT4^+^ cells per mm^2^, pre-spermatogonia as the number of MAGE-A4^+^ cells per mm^2^ and total germ cell number as the combined number of OCT4^+^ and MAGE-A4^+^ cells per mm^2^. Values represent mean ± SEM, with *N*=5 (vehicle control), *N*=5 (flutamide). Significant difference compared to vehicle control *p<0.05

To validate that the effects observed in ketoconazole-treated fetal testis from GW 7–12 were the result of reduced androgen action, the effects of flutamide (which blocks signalling through the androgen receptor [24]) were examined in human fetal testis. Cultured tissue samples (GW 7–12) were treated with flutamide (10^−6^ M) for 2 weeks. Flutamide treatment did not alter the production of androgens (testosterone, androstenedione and DHEAS) compared to vehicle control treated samples (Additional file 3: Fig. S3A-C), while treatment with ketoconazole (10^−6^ M) in these fetal samples resulted in the expected reduction in androgen production, including testosterone (42%, *p*<0.0001), androstenedione (34%, *p*<0.05) and DHEAS (40%, *p*<0.05) (Additional file 3: Fig. S3). Also, flutamide treatment did not affect testicular morphology, seminiferous cord structure or apparent expression of the proliferation marker (BrdU), apoptosis marker (cPARP) or the expression of CYP11A1 or AR (Additional file 4: Fig. S4). Interestingly, treatment with flutamide resulted in reduced secretion of INSL3 (38%, *p*<0.05) as well as AMH (37%, *p*<0.05) and Inhibin B (55%, *p*<0.01) (Fig. 8A–C). Also, ketoconazole treatment resulted in reduced secretion of INSL3 (41%, *p*<0.001), AMH (26%, *p*<0.05) and Inhibin B (72%, *p*<0.001) in this series of experiments (Fig. 8A–C). Treatment with flutamide did not affect the density of OCT4^+^ gonocytes or total germ cell density, but the density of pre-spermatogonia determined as MAGE-A4^+^ cells/mm^2^ was reduced (66%, *p*<0.05) (Fig. 9A–C). Treatment with ketoconazole in GW 7–12 samples did not alter the density of gonocytes, pre-spermatogonia or total germ cells (Fig. 9A–C).

## Discussion

The hypothesis that sufficient androgen exposure of the human fetal testis during a presumptive androgen sensitivity period is essential for the programming of male reproductive function throughout life is intriguing. Despite the identification of MPW in rats more than a decade ago [14] and the suggestion of a human equivalent [14, 25], experimental evidence for such a presumptive human MPW has not been presented. In the present study, we provide experimental evidence to support the presence of an androgen-sensitive window during human fetal testis development. This was identified after experimentally reducing the androgen production in ex vivo cultured human fetal testis tissue from 1^st^ and 2^nd^ trimesters in an established model [12, 18, 19] and examining the effects on cellular composition, germ cell maturation and testicular function. The ketoconazole-mediated decrease in androgen production resulted in reduced secretion of the Leydig cell factor INSL3 as well as the Sertoli cell factors AMH and Inhibin B in the age groups GW 7–10 and 10–12, but not GW 12–16 and 16–21. Additionally, a reduced density of germ cells (gonocytes) was found in the GW 7–10 age group. These findings were subsequently validated in GW 7–12 human fetal testis ex vivo following the reduction of androgen action via blocking of the androgen receptor by flutamide treatment. The flutamide-mediated reduced androgen exposure also resulted in reduced secretion of INSL3, AMH and Inhibin B as well as a decreased density of pre-spermatogonia. Thus, the overlap in effects observed after ketoconazole and flutamide treatments in human fetal testes suggests that these are indeed the result of experimentally reducing androgen exposure through different modes of action.

The consistent differences in effects between the examined age groups following ketoconazole-mediated reduction in androgen production were an important finding indicating (1) the presence of an androgen-sensitive period during human fetal testis development and (2) that the human androgen-sensitive window of testis development lies between GW 7–14 (including the 2-week culture period). Both notions are in accordance with previous suggestions that a human equivalent of the rat MPW exists. Extrapolations from the timing of the MPW in rats to the human fetal developmental timeline suggest that it lies between GW 8–14 [14, 25]. This androgen-sensitive window during human testis development may similarly program testicular function later in fetal and/or postnatal life, although it was not possible to examine this in the ex vivo culture model of fetal testis used in this study.

Ketoconazole inhibits the activity of several CYP enzymes involved in steroidogenesis resulting in a decreased production of androgens [26, 27]. Thus, the reduced androgen secretion reported in all examined age groups in this study was in accordance with the expected effect of ketoconazole. Additionally, the ketoconazole-mediated effects found in the age groups GW 7–10 and 10–12 in the present study were overall in accordance with the results previously reported. Using similar types of ex vivo culture approaches for human fetal testis, several studies have examined the effects of ketoconazole [28–30]. In a study examining the effects of ketoconazole (10^−5^ M) on human fetal testis from GW 7–12, a reduced production of testosterone, INSL3 and AMH as well as disruption of cord structures was reported [28]. The 10-fold higher dose of ketoconazole may explain the effect on cord structure since the same group in a subsequent study determined EC_50_ for ketoconazole-mediated reduction in testosterone production to be ~10^−6^ M and found no effects on cord structure, apoptosis or expression of CYP11A1 following treatment with this dose [29]. In the present study, no effects on cord structure, expression of examined somatic cell lineage markers or apparent changes in the density of proliferating (BrdU^+^) or apoptotic (cPARP^+^) cells were evident, indicating that the 10^–6^ M treatment dose did not promote cytotoxic effects. This is also in line with results from ex vivo culture of the human fetal testis (GW 7–12) treated with a 4-fold higher dose than the present study where no effect on cord structure or cytotoxicity was reported [29]. Although the selected 10^–6^ M ketoconazole dose used in the present study resulted in significantly reduced androgen production in all four age groups, a more pronounced effect was observed in the GW 7–10 and GW 10–12 groups compared to GW 13–16 and GW 17–21 groups. This may be the result of the higher level of testosterone production expected in GW 12–16 fetal testis [31]. Thus, it is not possible to exclude that a higher dose of ketoconazole would have been needed to ensure a similar level of reduction in androgen biosynthesis in the 2^nd^-trimester ex vivo cultured samples. However, the limited access to human fetal testis tissue did not allow for experiments with several different doses of ketoconazole in the present study and thus a single dose was used for all age groups since it provided the best opportunity for direct comparison between groups. The flutamide-mediated effects reported here cannot be directly compared to previous ex vivo culture studies on human fetal testes. However, in vitro studies have reported IC_50_ values ranging from 100 nM to 10 µM in androgen receptor assays with several studies reporting IC_50_ values around 10^−6^ M [32–34]. Additionally, the effects of 10^−7^ M flutamide were recently reported in a bovine in vitro granulosa cell culture model [35].

The reduced androgen exposure during the presumptive androgen-sensitive window of human fetal testis development identified in this study consistently resulted in reduced secretion of AMH and Inhibin B from the Sertoli cells which may be important for the overall fetal testis development and function. Sertoli cells in the fetal testes are involved in supporting germ cell survival and differentiation as well as promoting differentiation of fetal Leydig cells and support of the precursor cells of adult Leydig cells. AMH secretion is essential for masculinization and is normally high during the fetal and neonatal period, despite the high levels of intratesticular androgens, which in postnatal (peripubertal) testes are thought to suppress the AMH expression [36]. This has been attributed to the lack of androgen receptor (AR) expression in fetal Sertoli cells [37, 38], and the data in the current study confirm this pattern. The reduced AMH levels found after the ketoconazole-mediated reduction in androgen production in the present study were in accordance with the effects reported previously in the study by Mazaud-Guittot [28]. Since no AR expression was found in Sertoli cells in the examined developmental period, it is not clear by which mechanism the reduced androgen exposure resulted in the suppression of AMH, and, conversely, how the hCG-induced increase in androgen levels resulted in elevated levels of AMH. There is a possibility that PTM cells, which are observed within the developing fetal testis from GW 12 [39] and express AR, could be involved. The PTM cells transmit signals between Leydig cells and Sertoli cells, which may in part explain the different responses observed before/after ~GW 12 following manipulation of androgen production in the present study. Additionally, it cannot be excluded that the reduced androgen levels following ketoconazole and flutamide treatment could have affected the somatic niche in a manner that was not distinguishable based on tissue morphology and expression of somatic cell lineage markers, e.g. reduced the density and/or development of Sertoli cells, or secretion of other factors not measured in the present study. Although the expression pattern of the Sertoli cell marker SOX9 showed no apparent change following any of the treatments, there may be a slight reduction in the density of Sertoli cells following ketoconazole treatment in the GW 7–10 and GW 10–12 groups which could in part explain the observed reduced levels of AMH and Inhibin B. Also, it is not evident from our results whether the reduced levels of INSL3 could be the result of reduced AMH and Inhibin B levels or vice versa. The reduced secretion of INSL3 consistently observed after ketoconazole and flutamide-mediated reduction in androgen exposure during the androgen-sensitive window may suggest an additional direct effect on the fetal Leydig cells that express AR throughout the examined developmental period, but the precise regulation and function of INSL3 in the human fetal testis is not understood in detail. However, in vivo INSL3 together with testosterone promotes testicular descent, and reduced levels of INSL3 in cord blood at birth have been associated with cryptorchidism [40, 41]. The reduced secretion of AMH and Inhibin B may explain the observed effects on germ cell density, which also decreased after ketoconazole- and flutamide-mediated reduced androgen exposure during the androgen-sensitive period (GW 7–12). Interestingly, the affected germ cell type (gonocytes vs. pre-spermatogonia) was not consistent between treatments, which either may be due to direct differential effects (modes of action) of ketoconazole and flutamide treatment or could be the result of altered secretion of other Sertoli cells factors that were not determined in the present study (DHH, Activin B, FGF9). Regardless, this observation may have implications for the establishment of the spermatogonial stem cell population which is essential for future spermatogenesis and fertility. However, this possible effect remains speculative due to the short-term ex vivo culture approach in the present study.

Overall, the use of an ex vivo tissue culture model of isolated human fetal testes in the present study warrants a cautious interpretation of the reported findings since it does not allow the determination of effects on other organs or the impact of endocrine feedback mechanisms. Therefore, it is not possible to directly translate effects from the ex vivo culture model into an in vivo situation nor to provide insight about the development of male reproductive disorders that manifest later in life. The exclusion of hCG from the basic culture media contrasts with the in vivo situation where hCG is continuously present. However, the addition of hCG to culture media resulted in a lack of significant androgen reduction after ketoconazole treatment and since the main purpose of these experiments was to reduce androgen production, the experimental approach with either ketoconazole or hCG treatment was selected for this study. Of note, the culture media composition has been optimized prior to the establishment of the ex vivo culture set-up and used in previous studies [12, 18, 19]. Another important limitation of the experimental approach in the present study is that it did not allow the detection of possible transient effects of treatments on hormone levels in culture media, which were pooled for each tissue fragment throughout the culture period due to the small volume. Also, transient effects on specific cell populations might not be detected since the tissue is analysed at the end of the 2-week culture period. Thus, it is not possible to exclude transient effects of ketoconazole or flutamide treatment in the present study. Finally, since experiments included in this study were performed in two different laboratories: Copenhagen (fetuses aged GW 7–12) and Edinburgh (fetuses aged GW 13–22), it is not possible to exclude the possibility that minor differences in handling and culture of tissue could have affected the results—although it is important to emphasize that all experiments were performed in a manner where tissues from each individual fetus were subjected to control and treatment under the same conditions and subsequently analysed as paired samples. Thus, the findings of the present study are in line with previous results from animal models. Our study provides novel insights into the existence and timing of a distinct androgen-sensitive period for programming of somatic cell function in the human fetal testis that coincides with the timing of the MPW identified in the rat, which is also associated with somatic cell dysfunction.

## Conclusions

This study suggests the presence of a human window of androgen sensitivity in the testis that lies between GW 7–14. Experimentally reduced androgen action during this period affects the function of fetal Sertoli and Leydig as well as the density of germ cells which may have implications for the development of the androgen-sensitive tissues and for testicular function later in life.

## Supplementary Information

**Additional file 1:** **Figure S1.** Effects of manipulating androgen production or androgen exposure via blocking of the androgen receptor in *ex vivo *culture of human fetal testes. Expression of the proliferation marker (BrdU) and apoptosis marker (cPARP) in fetal testis samples treated with ketoconazole (10^-6^ M) and flutamide (10^-6^ M) for two weeks in *ex vivo* culture. Images representative for the expression in samples aged GW 7-12. Counterstaining with Mayer haematoxylin, scale bar corresponds to 50 µm.

**Additional file 2:** **Figure S2.** Effects of manipulating androgen production in *ex vivo* cultured human fetal testis tissue on expression of the Sertoli cell marker SOX9. Expression pattern of the Sertoli cell marker SOX9 in fetal testis samples treated with ketoconazole (10^-6^ M) and hCG (1 IU/ml) for two weeks in *ex vivo* culture. Images representative for the expression in each age-group and treatment. Counterstaining with Mayer haematoxylin, scale bar corresponds to 50 µm.

**Additional file 3:** **Figure S3.** Effects of reduced androgen exposure via blocking of the androgen receptor in *ex vivo* culture of human fetal testes. Quantification of **A)** testosterone **B)** androstenedione and **C)** DHEAS secretion by *ex vivo* cultured fetal testis tissue from GW 7-12 following treatment with flutamide (10^-6^ M) and ketoconazole (10^-6^ M) for 14 days**. **Media were collected every 48 hours throughout the 14-day culture period and were pooled for each individual tissue piece. Androgens were measured by LC-MS/MS and are shown as ratios compared to the mean of the corresponding vehicle controls (from the same fetus). Values represent mean ± SEM, with *N*=13-15 (vehicle control),*N*=7-9 (flutamide) and *N*=9-10 (ketoconazole). Significant difference compared to vehicle control, ****P<0.0001, * *P*<0.05.

**Additional file 4:** **Figure S4.** Effects of reduced androgen action via blocking of the androgen receptor in *ex vivo* culture of human fetal testes. Expression pattern of CYP11A1 and androgen receptor (AR) in fetal testis samples treated with flutamide (10^-6^ M) for two weeks in *ex vivo* culture. Images representative for the expression in samples aged GW 7-12. Counterstaining with Mayer haematoxylin, scale bar corresponds to 50 µm.

## Data Availability

All data generated or analysed during this study are included in this published article and supplementary material or are available from the corresponding author upon request.

## Abbreviations

17-OHP: 17-Hydroxyprogesterone
AR: Androgen receptor
DHEAS: Dehydroepiandrosterone sulphate
GW: Gestational week
IHC: Immunohistochemistry
MPW: Masculinization programming window
PTM: Peritubular myoid cell
RSD: Relative standard deviation
SCO: Sertoli cell only
TBS: Tris-buffered saline
TDS: Testicular dysgenesis syndrome
