# Supplementary figures and images for: Identification of a window of androgen sensitivity for somatic cell function in human fetal testis cultured ex vivo
Source: BMC Med. 2022 Oct 20;20:399. doi: 10.1186/s12916-022-02602-y (PMC9585726; doi:10.1186/s12916-022-02602-y)

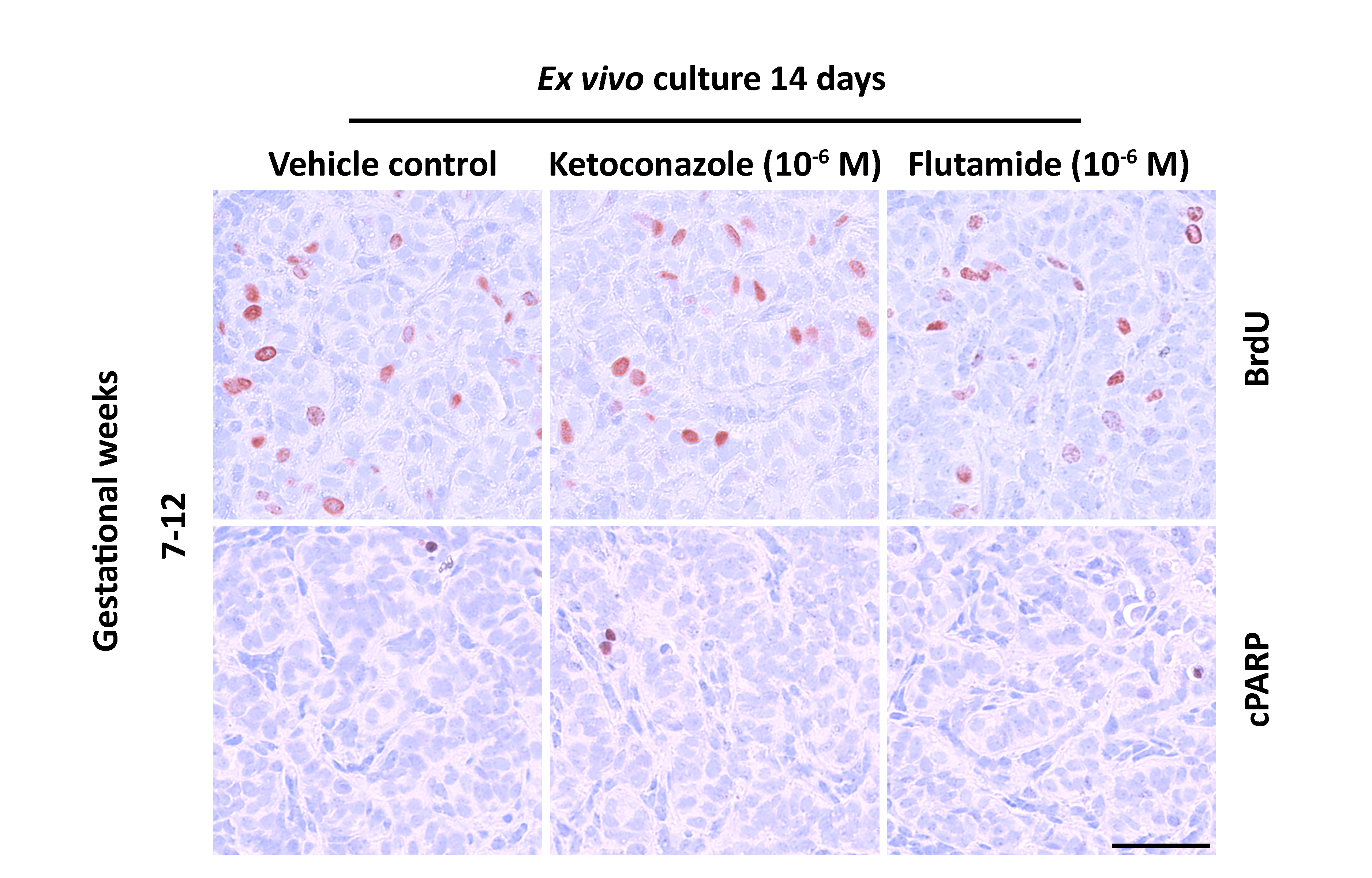

Supplement: Supplementary file 1 — Additional file 1: Figure S1. Effects of manipulating androgen production or androgen exposure via blocking of the androgen receptor in ex vivo culture of human fetal testes. Expression of the proliferation marker (BrdU) and apoptosis marker (cPARP) in fetal testis samples treated with ketoconazole (10-6 M) and flutamide (10-6 M) for two weeks in ex vivo culture. Images representative for the expression in samples aged GW 7-12. Counterstaining with Mayer haematoxylin, scale bar corresponds to 50 µm. [file 12916_2022_2602_MOESM1_ESM.tif]

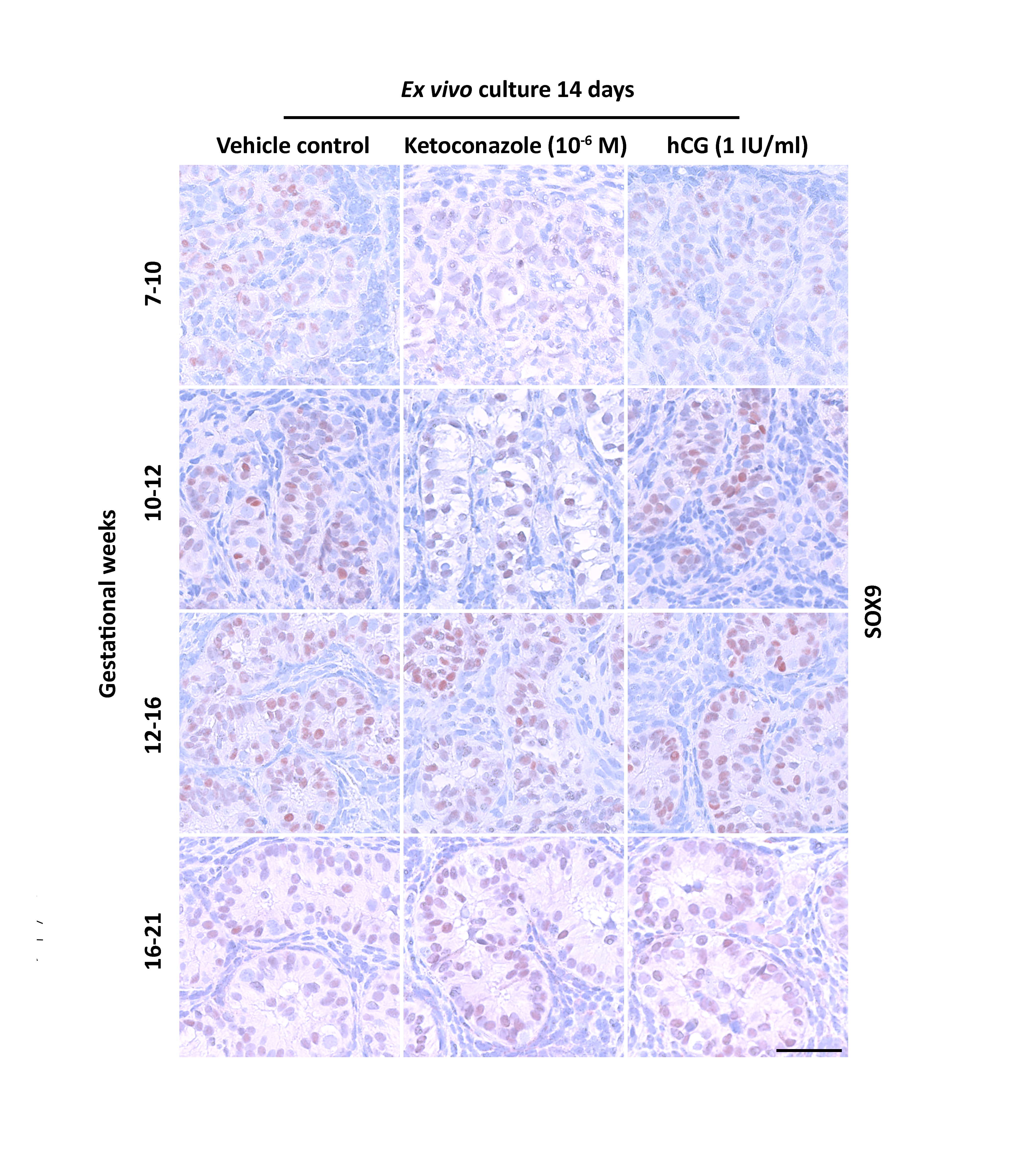

Supplement: Supplementary file 2 — Additional file 2: Figure S2. Effects of manipulating androgen production in ex vivo cultured human fetal testis tissue on expression of the Sertoli cell marker SOX9. Expression pattern of the Sertoli cell marker SOX9 in fetal testis samples treated with ketoconazole (10-6 M) and hCG (1 IU/ml) for two weeks in ex vivo culture. Images representative for the expression in each age-group and treatment. Counterstaining with Mayer haematoxylin, scale bar corresponds to 50 µm. [file 12916_2022_2602_MOESM2_ESM.tif]

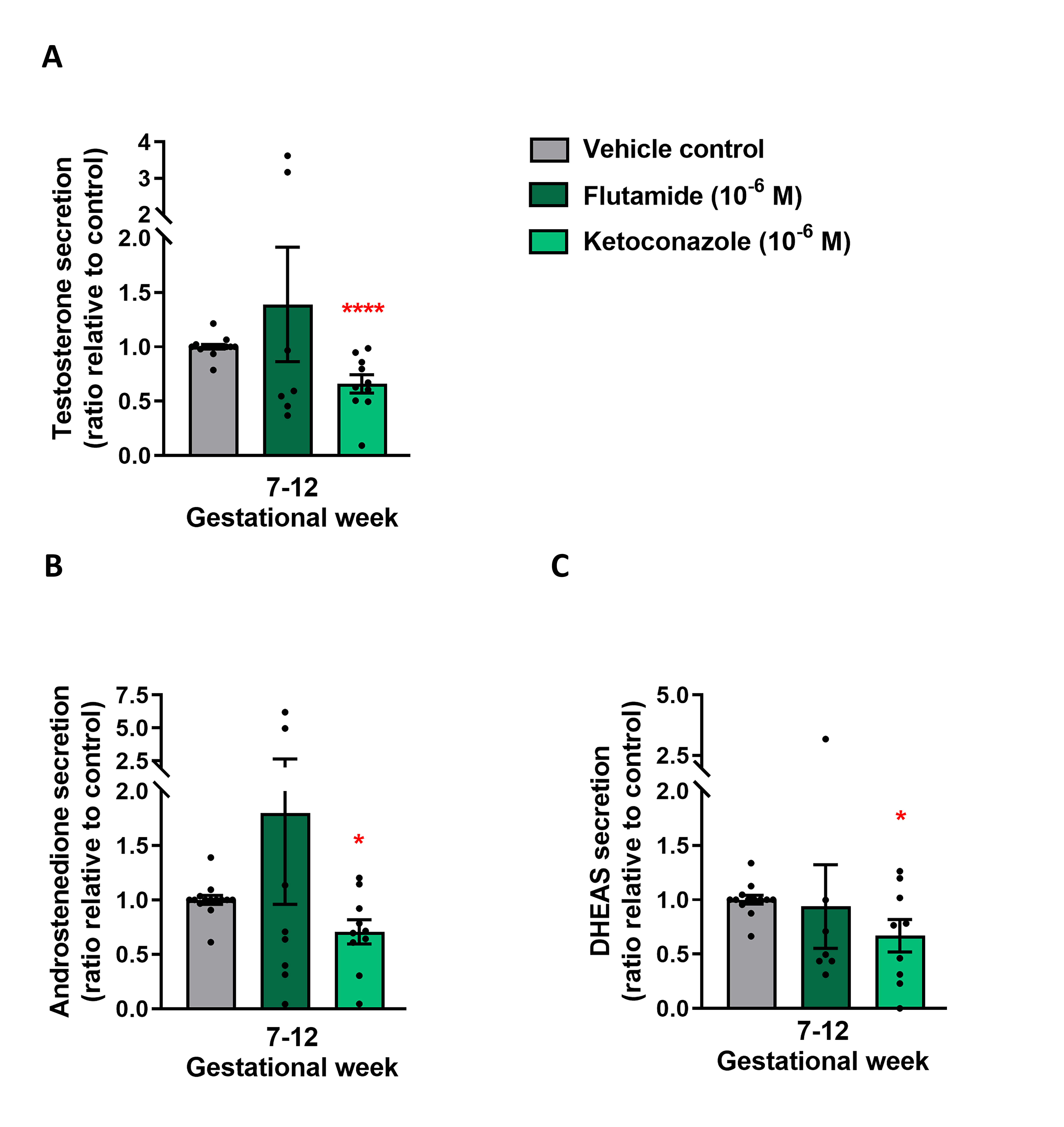

Supplement: Supplementary file 3 — Additional file 3: Figure S3. Effects of reduced androgen exposure via blocking of the androgen receptor in ex vivo culture of human fetal testes. Quantification of A) testosterone B) androstenedione and C) DHEAS secretion by ex vivo cultured fetal testis tissue from GW 7-12 following treatment with flutamide (10-6 M) and ketoconazole (10-6 M) for 14 days. Media were collected every 48 hours throughout the 14-day culture period and were pooled for each individual tissue piece. Androgens were measured by LC-MS/MS and are shown as ratios compared to the mean of the corresponding vehicle controls (from the same fetus). Values represent mean ± SEM, with N=13-15 (vehicle control),N=7-9 (flutamide) and N=9-10 (ketoconazole). Significant difference compared to vehicle control, ****P<0.0001, * P<0.05. [file 12916_2022_2602_MOESM3_ESM.tif]

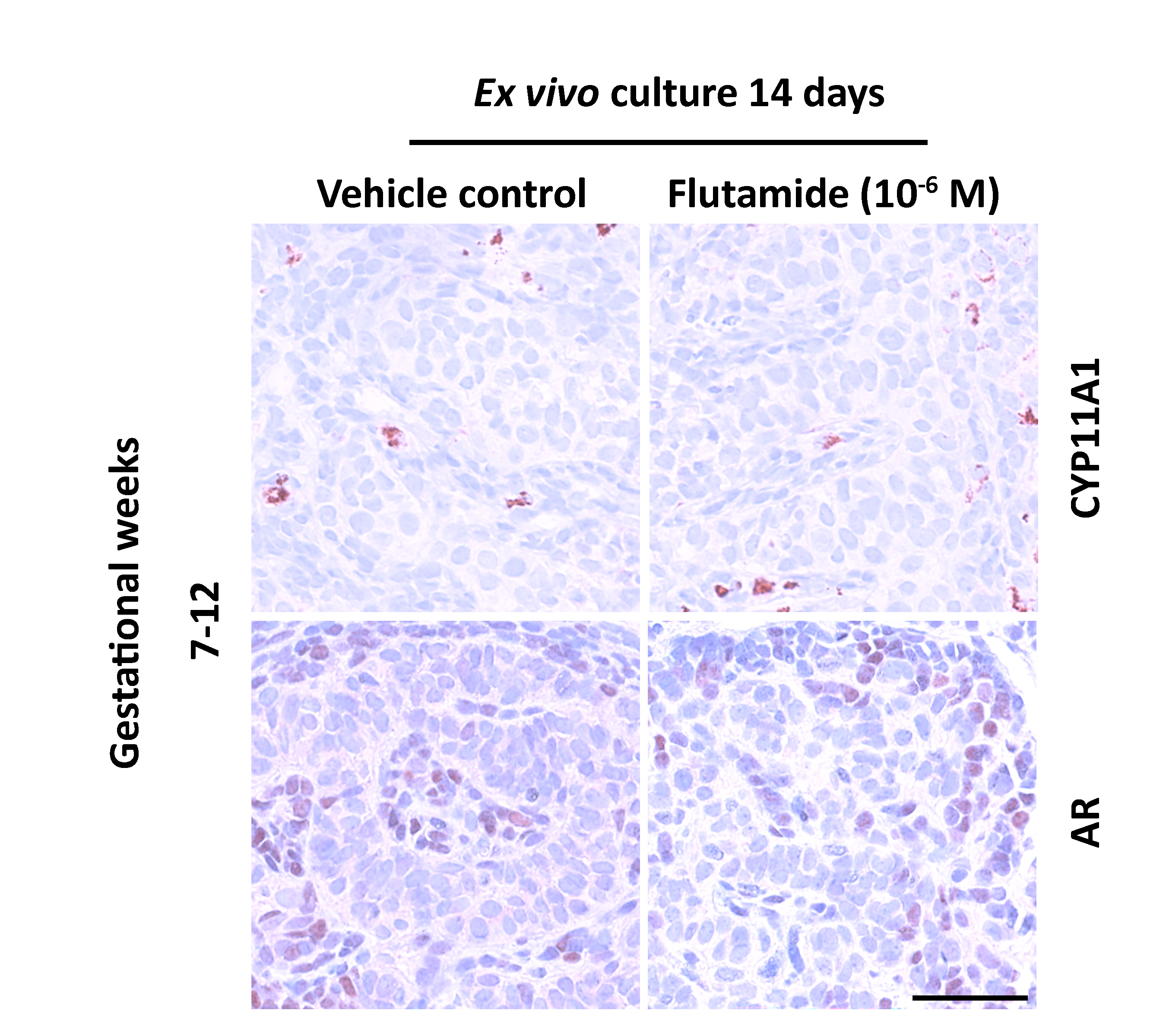

Supplement: Supplementary file 4 — Additional file 4: Figure S4. Effects of reduced androgen action via blocking of the androgen receptor in ex vivo culture of human fetal testes. Expression pattern of CYP11A1 and androgen receptor (AR) in fetal testis samples treated with flutamide (10-6 M) for two weeks in ex vivo culture. Images representative for the expression in samples aged GW 7-12. Counterstaining with Mayer haematoxylin, scale bar corresponds to 50 µm. [file 12916_2022_2602_MOESM4_ESM.tif]
